# Regulation of laboratory-developed tests and in-house *in vitro* diagnostic medical devices in the United States and the European Union—a comparative overview

**DOI:** 10.1016/j.esmoop.2025.105909

**Published:** 2025-11-26

**Authors:** A. Kahles, A.-L. Volckmar, H. Goldschmid, M. Salto-Tellez, M. Vogeser, J. Stone, M. Brüggemann, J. Budczies, D. Kazdal, R. Salgado, P. Schirmacher, S. Peters, J.K. Lennerz, A. Stenzinger

**Affiliations:** 1 Institute of Pathology, University Hospital Heidelberg, Heidelberg, Germany; 2 Precision Medicine Centre, Queen’s University Belfast, Belfast, UK; 3 Institute of Laboratory Medicine, LMU University Hospital, Ludwig-Maximilians-University Munich, Munich, Germany; 4 Mass Spectrometry & Advances in the Clinical Lab-Compliance and Accreditation Committee, Del Mar, USA; 5 2nd Internal Medicine Department, Hematology Lab Kiel, University Hospital Schleswig-Holstein (UKSH), Kiel, Germany; 6 Department of Pathology, ZAS Hospitals, Antwerp, Belgium; 7 Division of Research, Peter Mac Callum Cancer Centre, Melbourne, Australia; 8 Medical Oncology, Oncology Department – CHUV Lausanne University, Lausanne, Switzerland; 9 BostonGene Corporation, Waltham, USA

**Keywords:** laboratory developed tests (LDT), in-house *in vitro* diagnostic medical devices (IH-IVD), regulation, IVDR, FDA, legislation

## Abstract

Process chains in medical diagnostic laboratories lead to an accurate diagnosis and consequently to optimized personalized therapy recommendations. In addition to approved commercial *in vitro* diagnostic medical devices, devices and tests manufactured and used within a single diagnostic laboratory play a decisive role in these process chains ensuring state-of-the-art diagnostics and consecutive best patient care.

It is vital that the implementation and use of such in-house tests, processes and medical devices developed in diagnostic laboratories by health care professionals is quality assured and meets regulatory and legal requirements. Due to the complexity and variety of these tests as well as their dual use in routine care and clinical trials, a thorough understanding of these regulations and their underlying definitions is critical.

This review provides a comparative overview of the current state of the regulation of laboratory-developed tests in the United States and in-house developed *in vitro* diagnostic medical devices in the European Union. It dissects and compares the relevant regulatory ecosystems to identify conceptual similarities and differences. Our work helps laboratories navigate the regulatory landscape and provides a basis for further discussions among key stakeholders including health care providers, payers, legislators and regulatory agencies.

## Highlights

• *In vitro* diagnostics play a crucial role in medical laboratories for accurate diagnoses and therapy recommendations.
• Development and use of in-house tests and devices require quality assurance to meet regulatory and legal requirements.
• This review provides a comparative overview of USA and EU regulations on LDTs and in-house IVDs.
• Our work helps laboratories navigate the regulatory landscape and informs discussions among key stakeholders.
• Future policy decisions should prioritize clarity, proportionality and coherence to protect innovation and patient safety.

## Introduction

In diagnostic laboratories, validated process chains lead to a reliable diagnosis supporting patient management. Examinations in specialized diagnostics are generally characterized by multi-step, highly complex and quality assured processes for which physicians are responsible.

In addition to approved commercial *in vitro* diagnostic medical devices (IVDs), such as CE-marked IVDs (*‘conformité européenne’*, CE-IVDs) in the European Union (EU) or IVDs approved by the Food and Drug Administration (FDA) in the USA in-house developed IVDs (IH-IVDs) and laboratory-developed tests (LDT) play a decisive role in diagnostics workflows, ensuring innovative and rapidly implementable state-of-the-art diagnostics and optimal patient care. Due to their nature, both LDTs and IH-IVDs often fill gaps in the diagnostic market, improve the breadth and depth of the test choices, and/or deliver testing in a significantly more affordable fashion. Importantly, such tests may also be used in clinical trials. The term IH-IVD is commonly used in Europe, whereas LDT or laboratory-developed testing procedure (LDP) are the standard terms in the USA. In both the EU and the USA, it is essential that the implementation and use of in-house tests, in-house processes and IVDs developed in diagnostic laboratories by health care professionals is quality assured and follows regulatory and legal requirements.

According to EU Treaty Article 168, the regulation of processes in medical care lies with the 27 member states. However, the manufacture and use of IVDs (including IH-IVDs) has been managed by Regulation (EU) 2017/746 on *in vitro* diagnostic medical devices (EU-IVDR) since 2017. In the USA, in-laboratory processes and LDTs are the realm of the Centers for Medicare & Medicaid Services (CMS) through the Clinical Laboratory Improvement Amendments (CLIA) program. In this context it is important to highlight that the FDA has attempted to gain oversight of LDTs through a Final Rule in 2024 (with an anticipated go-live date in May 2025) but the rule was recently vacated and set aside by a final United States District Court ruling following a consolidated lawsuit filed by the American Clinical Laboratory Association (ACLA) and the Association for Molecular Pathology (AMP).^1^^,^^2^

Here, we provide a comparative overview of the current regulation of LDTs in the USA and IH-IVDs in the EU. We outline the underlying regulatory motivations and considerations, highlight relevant regulatory similarities and differences, and present the regulatory efforts in terms of their objectives. In line with the complex and diverse nature of LDTs and IH-IVDs, their legal definition and therefore their regulation is complex (Table 1). The lack of clarity in existing mandates, guidance and oversight makes it difficult for laboratories to navigate compliance requirements, increasing both their workload and overall uncertainty. This overview is intended to help laboratories better understand and prepare for this evolving regulatory landscape. Moreover, we present a basis for further discussions among key stakeholders, including health care providers, payers, legislators and regulatory agencies.

**Table 1:** Definitions according to different legislators, administration and associations

Term	Definitions	Reference
**Europe**		
**Device**	For the purposes of this Regulation, *in vitro* diagnostic medical devices and accessories for *in vitro* diagnostic medical devices shall hereinafter be referred to as ‘devices’.	Definition by Regulation [European Union (EU)] 2017/746 on IVDs (EU-IVDR)^3^ Article 1 (2)
***In vitro* diagnostic medical device (IVD)**	‘*In vitro* diagnostic medical device’ means any medical device which is a reagent, reagent product, calibrator, control material, kit, instrument, apparatus, piece of equipment, software or system, whether used alone or in combination, intended by the manufacturer to be used *in vitro* for the examination of specimens, including blood and tissue donations, derived from the human body, solely or principally for the purpose of providing information on one or more of the following: (a) concerning a physiological or pathological process or state; (b) concerning congenital physical or mental impairments; (c) concerning the predisposition to a medical condition or a disease; (d) to determine the safety and compatibility with potential recipients; (e) to predict treatment response or reactions; (f) to define or monitoring therapeutic measures. specimen receptacles shall also be deemed to be *in vitro* diagnostic medical devices.	Definition by Regulation (EU) 2017/746 on IVDs (EU-IVDR)^3^ Article 2 (2)
**Health institution**	‘Health institution’ means an organisation the primary purpose of which is the care or treatment of patients or the promotion of public health.	Definition by Regulation (EU) 2017/746 on *in vitro* diagnostic medical devices (EU-IVDR)^3^ Article 2 (29)
**In-house device**	A device that is manufactured and used only within a health institution established in the Union and that meets all conditions set in Article 5 (5) of the […] IVDR.	Definition by Medical Device Coordination Group according to article 99, IVDR; Document MDCG-2023-01^4^
**USA**		
**LDTs**	‘Laboratory developed tests’, or LDTs, are IVDs that are intended for clinical use and designed, manufactured, and used within a single clinical laboratory that is certified under the Clinical Laboratory Improvement Amendments of 1988 (CLIA) and meets the regulatory requirements under CLIA to perform high complexity testing.’	Definition by United States Food and Drug Administration (FDA)^5^
**IVDs**	‘IVDs’ are devices under the Federal Food, Drug, and Cosmetic Act (FD&C Act) including when the manufacturer of the IVD is a laboratory. IVDs are intended for use in the collection, preparation, and examination of specimens taken from the human body, such as blood, saliva, or tissue. IVDs, including LDTs, can be used to measure or detect substances or analytes in the body, such as proteins, glucose, cholesterol, or DNA, to provide information about a patient’s health, including to diagnose, monitor, or determine treatment for diseases and conditions.’	Definition by United States FDA^5^
**LDPs**	‘Laboratory-developed testing procedures (LDPs)’ are services that hospitals and academic and clinical laboratories develop and use in patient care. These services are not commercially manufactured and marketed but are designed, developed, validated, performed and interpreted by board-certified professionals in a single laboratory. (Regulated by CLIA)	Definition by Association for Molecular Pathology^6^
**Device**	Per Section 201(h) (1) of the Food, Drug, and Cosmetic Act, a device is: An instrument, apparatus, implement, machine, contrivance, implant, *in vitro* reagent, or other similar or related article, including a component part, or accessory which is: (A) recognized in the official National Formulary, or the United States Pharmacopoeia, or any supplement to them, (B) intended for use in the diagnosis of disease or other conditions, or in the cure, mitigation, treatment, or prevention of disease, in man or other animals, or (C) intended to affect the structure or any function of the body of man or other animals, and which does not achieve its primary intended purposes through chemical action within or on the body of man or other animals and which is not dependent upon being metabolized for the achievement of its primary intended purposes. The term ‘device’ does not include software functions excluded pursuant to section 520(o).	Section 201(h) (1) of the Food, Drug, and Cosmetic Act^7^

## Regulation in the united states

### Current legal framework for LDTs in the USA

**Figure 1:**
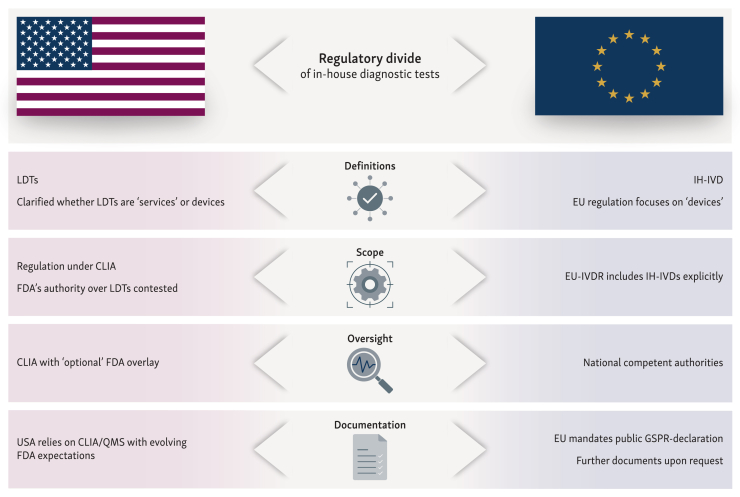
**Key regulatory divergences of in-house diagnostic tests (USA LDTs versus EU IH-IVDs).** CLIA, Clinical Laboratory Improvement Amendments; EU, European Union; EU-IVDR, Regulation (EU) 2017/746 on *in vitro* diagnostic medical devices; FDA, Food and Drug Administration; GSPR, general safety and performance requirements (Annex I, EU-IVDR); IH-IVD, in-house *in vitro* diagnostic medical devices; LDT, laboratory-developed test; QMS, quality management system.

In the USA, LDTs are regulated by the CMS through the CLIA program (Figure 1, Table 2). The CLIA governs laboratories that test human specimens to ensure accurate and reliable results.^8^^,^^9^ Before an LDT can be used for patient care, CLIA requires laboratories to establish performance specifications related to analytical validity within their specific testing environment. These are typically reviewed during routine biennial inspections, including personal competency. LDTs are classified as high-complexity tests under CLIA, as these tests pose more than minimal risk to patients in the event of a false result.

**Table 2:** Comparative overview of the regulation of laboratory-developed tests (LDT) and in-house *in vitro* diagnostic medical devices (IH-IVD) in the United States and the European Union (EU)

Regulatory parameters	EU-IVDR, article 5(5) and annex I^3^ (in force)	CMS/CLIA^9^ (in force)	FDA’s Final Rule^14^ (vacated and set aside by judgment)
Quality management system	Manufacture and use of IH-IVDs occur under appropriate quality management systems. The laboratory of the health institution is compliant with standard EN ISO 15189 ‘Medical laboratories - requirements for quality and competence’ or where applicable national provisions, including national provisions regarding accreditation.	Laboratories must meet the requirements under the Clinical Laboratory Improvement Amendments (CLIA) which regulates the quality and safety of USA clinical laboratories to ensure the accuracy, reliability and timeliness of patient test results regardless of where the test was performed. Under CLIA, LDTs are classified as high-complexity tests. As high-complexity tests, LDTs must adhere to more stringent requirements before being used on patient samples [e.g. 42 CFR Part 493.1253(b) (1)].	Laboratories should comply with the harmonized Quality Management System Regulation (QMSR) that includes both the FDA Quality System Regulation (QSR) requirements and the requirements of ISO 13485 ‘Medical devices – Quality management systems – Requirements for regulatory purposes requirements’. Laboratories should incorporate certain elements of current good manufacturing practices (cGMP) with their quality management system.
Risk classification	EU-IVDR describes seven classification rules for risk classes A, B, C and D (annex VIII). The greater the risk of a life-threatening disease and the larger the group of people affected, the higher the risk class. - Class A: low individual and low public risk - Class B: moderate individual and low public risk - Class C: high individual and moderate public risk - Class D: high individual and high public risk	The CLIA requirements are based on the test complexity; the more complex the test is to perform, the more stringent the requirements. LDTs are considered high-complexity tests. Therefore, the laboratory must meet all applicable CLIA requirements for high-complexity testing. Tests with high complexity can only be performed in a laboratory that is certified as a high-complexity laboratory under CLIA.	FDA classified LDTs based on the intended use of the test and risk posed to patients. Class I: low-risk LDTs with minimal risk for harm. Class II: moderate-risk LDTs that fall in a mid-range risk for harm. Class III: high-risk LDTs that support or sustain human life, which presents a potential for unreasonable risk of harm.
Risk management	Risk management is part of the general safety and performance requirements (GSPR) set out in annex I and shall be understood as a continuous iterative process throughout the entire lifecycle, requiring regular systematic updating. The GSPR lists several requirements for the mandatory IVD specific risk management.	Not explicitly stated, but many elements are included in CLIA quality assurance regulation that mitigate risk. So, risk is indirectly addressed through a focus on various areas. - personnel qualification - proficiency testing - quality control procedures - evaluation of test performance - corrective actions when errors are detected	Risk management was a core component of the FDA’s proposed framework. Laboratories were required to establish and maintain procedures to identify potential hazards, assess associated risks, implement control measures and monitor effectiveness throughout the test’s lifecycle. This aligned with ISO 14971 principles for medical devices, emphasizing a proactive and documented approach to managing patient safety and test reliability.
Performance evaluation/validation	Evaluation of performance characteristics is required. In particular and where applicable: Analytical performance: such as, analytical sensitivity, analytical specificity, trueness (bias), precision (repeatability and reproducibility), accuracy (resulting from trueness and precision), limits of detection and quantitation, measuring range, linearity, cut-off, including determination of appropriate criteria for specimen collection and handling and control of known relevant endogenous and exogenous interference, cross-reactions. Clinical performance: such as diagnostic sensitivity, diagnostic specificity, positive predictive value, negative predictive value, likelihood ratio, expected values in normal and affected populations.	Analytical validation required: includes an analysis of accuracy, precision, analytical sensitivity, analytical specificity, reportable range, reference interval, and any other performance characteristics required for the test system in the laboratory that intends to use it. Clinical validation: CLIA survey does not include a review of the clinical validation of an LDT. However, clinical validation is required by certain states (e.g. New York), and certain accreditation organizations [e.g. College of American Pathologists (CAP)], and relevant documentation may be reviewed during a CLIA inspection.	Analytical validation: FDA’s analytical validation is focused on accuracy, precision, analytical sensitivity and specificity, reportable range, and robustness. Clinical validation: Clinical validation was required to demonstrate that the test meaningfully predicts the clinical condition, outcome, or risk—based on intended use. A range of data types can satisfy the FDA’s clinical validity requirement, including supportive scientific literature, testing clinically defined patient specimens in comparison to a predicate device, and clinical trials. Both validations were assessed under a total product lifecycle approach, with documentation aligned to FDA’s expectations for safety and effectiveness.
Documentation	• Public declaration that used IH-IVDs meet the general safety and performance requirements (GSPR) set out in annex I. • Documentation that makes it possible to have an understanding of the manufacturing facility, the manufacturing process, the design and performance data of the devices, including the intended purpose, and that is sufficiently detailed to enable the competent authority to ascertain that the GSPR are met. • Justification that the target patient group’s specific needs cannot be met, or cannot be met at the appropriate level of performance by an equivalent CE-IVD. • Information on the use including a justification of the manufacturing, modification and use.	For all verification and validation activities, laboratories are required to maintain appropriate documentation for regulatory compliance and to ensure the quality of testing practices. CLIA requires written quality control procedures to monitor the accuracy and precision of the total resting process (pre-analytic, analytic, post-analytic), as well as to monitor the general laboratory systems.	Laboratories must submit premarket approval (PMA) applications for LDTs.
Registration	IH-IVDs must not be registered but the public declaration stating GSPR-conformity must name the details necessary to identify the IH-IVD.	LDTs are not required to register and list with FDA but laboratories performing LDTs must have a current CLIA certification which is registered.	Laboratories must register with the FDA as medical device manufacturers and list information about their LDTs in a public database.
Inspection/oversight	Competent authorities of EU member states: - authorities shall be permitted access to inspect the activities of the health institutions. - inspection of relevant documents upon request. - inspection of the activities of the health institution. - scope and periodicity of inspection and further details are regulated in the respective national legislations.	Every 2 years by CMS or ‘deemed status’ organizations. That means new LDTs can begin to be offered for patient testing before they are reviewed during inspection.	Oversight by FDA. Laboratories must submit PMA applications for LDTs.

This comparison contains selected parameters and is not intended to be exhaustive.

CMS, Centers for Medicare & Medicaid Services; FDA, Food and Drug Administration.

To accommodate the large number of laboratories requiring CLIA certification, CMS has the option of granting ‘deemed status’ to other organizations, allowing them to provide oversight on behalf of CMS [e.g. the College of American Pathologists (CAP) or the American Association for Laboratory Accreditation]. Successful accreditation by one of these organizations demonstrates that a laboratory is also compliant with CLIA requirements. Of note, many deemed status organizations have specific requirements in their accreditation standards that may go beyond CLIA requirements. For example, although CLIA does not require a review of clinical validity, this may be mandated by certain states (e.g. New York) or accreditation bodies such as the CAP.^10^

### FDA’s efforts toward stricter regulation and FDA oversight

From the FDA’s point of view, LDTs are IVDs and therefore considered devices under the Federal Food, Drug, and Cosmetic Act (FD&C Act) (Table 1). Under the FD&C Act, a device is conceptually defined as ‘an instrument, apparatus, implement, machine, contrivance, implant, *in vitro* reagent, or other similar or related article, including a component part, or accessory which is […] intended for use in the diagnosis of disease or other conditions, or in the cure, mitigation, treatment, or prevention of disease, in man […]’. The FDA defines LDTs as IVDs that are intended for clinical use and are designed, manufactured and used within a single laboratory that is CLIA certified and meets the regulatory requirements under CLIA to perform high-complexity testing. For this reason, the FDA is considering whether and to what extent it should assume specific responsibility for the regulation and monitoring of LDTs. However, until recently, the FDA has refrained from doing so, and—with its general enforcement discretion approach—the FDA has not directly taken on the oversight of LDTs itself. Therefore, LDTs do not require FDA premarket approval.^11^ However, as the FDA already indicated in its released ‘Framework for Regulatory Oversight of Laboratory Developed Tests’ in 2014 and in a subsequent discussion paper on LDTs in 2017, there is the overall intention to strengthen oversight of LDTs, to ensure risk-based control of these tests.^12^^,^^13^

With the FDA’s Proposed Rule published in October 2023 and its Final Rule published in May 2024, entitled ‘Medical Devices; Laboratory Developed Tests’, the FDA aimed to phase out its general enforcement discretion approach and bring LDTs under FDA oversight and approval under the FD&C Act in a modernized and unified regulatory framework to ensure safety and effectiveness to meet the increasing evolution and proliferation of LDTs.^11^^,^^14^^,^^15^ However, the rule was recently vacated and set aside by a final United States District Court ruling following a consolidated lawsuit filed by ACLA and AMP.^1^^,^^2^ According to this final judgment, the FDA does not have the authority to regulate LDTs as medical ‘devices’ due to the intangible nature of the medical professional service. Laboratories will not be required to comply with stage 1 of the FDA’s phased implementation of its Final Rule, which was scheduled to take effect in May 2025, nor with any subsequent stages and associated requirements. The FDA has officially declined to appeal the recent court ruling. This decision upholds the ruling that the FDA lacks the authority to impose premarket review requirements on LDTs/LDPs in the absence of explicit legislative guidance.

From a European perspective it is important to realize that this court ruling does not affect CLIA oversight, meaning that CLIA requirements for laboratories remain in effect. Hence, LDTs must continue to comply with CLIA regulations and, where applicable, with additional requirements set by a ‘deemed status’ accrediting organization before being used in patient care. However, following the court ruling, it can be assumed that new federal legislation will be required to establish a durable and comprehensive framework for the regulation of LDTs.

### Previous efforts toward stricter regulation

Following the FDA’s 2017 discussion paper, the Verifying Accurate Leading-edge IVCT Development (VALID) Act was introduced as a draft bill in 2018, with revised bipartisan versions brought before Congress in 2020, including referral to the Senate Health, Education, Labor and Pensions Committee. The core regulatory intent of the VALID Act was to unify the oversight of IVDs and LDTs as *in vitro* clinical tests (IVCTs), under a single risk-based FDA framework. VALID would impose FDA review and postmarket obligations in addition to the CLIA, particularly for moderate- and high-risk tests.^16^, ^17^, ^18^ Some low-risk IVCTs would be exempt or subject to technological certification pathways. Although the VALID Act was attached to the 2022 end-of-year omnibus spending package, the diagnostics provisions were ultimately excluded from the final legislation. Also, in 2020, a draft bill called the Verified Innovative Testing in American Laboratories (VITAL) Act was introduced in Congress. The VITAL Act proposed that oversight of LDPs remain solely under the CMS via the CLIA, explicitly limiting the FDA’s authority over such tests.^19^ However, like VALID, the VITAL Act has not been enacted into law.

## Regulation in the european union

According to EU Treaty Article 168, the regulation of processes in medical care lies with each of the 27 member states.^20^ In contrast, legislators appear to regard the regulation of IVDs’ access to the EU market as a task for the EU. Therefore, the manufacture and use of IVDs is regulated by the EU-IVDR, which aims at regulating both commercially available IVDs and IH-IVDs.

### EU-IVDR Regulation

Since 2017, the manufacture and use of IVDs have been regulated by the EU-IVDR.^3^ As an EU regulation, it is a valid and binding legal act in all member states. The aim of the EU-IVDR is to ensure a high level of health protection with a high level of patient and user safety. This is to be achieved through harmonized requirements governing the manufacture, trade, import and use of IVDs within the European market. The EU-IVDR basically distinguishes between two types of regulation-compliant IVDs: (i) CE-marked-IVDs, which are placed on the European market by economic operators following a conformity assessment procedure, and (ii) IH-IVDs. IH-IVDs are ‘manufactured’ by health institutions and may only be used within the manufacturing institution. Hence, the EU-IVDR establishes standardized requirements for IH-IVDs in the EU for the first time (Figure 1, Tables 2 and 3A). Though the regulation entered into force in May 2017, its requirements will be phased in from May 2022 to 2030 with various transition steps (Table 3A and B).

**Table 3:** A: EU-IVDR 2017/746 Article 5 (5),^3^ B: transitional provisions^21^^,^^22^

A| EU-IVDR 2017/746 - Article 5 (5)^3^
Chapter II: Making available on the market and putting into service of devices, obligations of economic operators, CE marking, free movement Article 5: Placing on the market and putting into service (5) With the exception of the relevant general safety and performance requirements set out in annex I, the requirements of this regulation shall not apply to devices manufactured and used only within health institutions established in the Union, provided that all of the following conditions are met: (a) the devices are not transferred to another legal entity; (b) manufacture and use of the devices occur under appropriate quality management systems; (c) the laboratory of the health institution is compliant with standard EN ISO 15189 or where applicable national provisions, including national provisions regarding accreditation; (d) the health institution justifies in its documentation that the target patient group’s specific needs cannot be met, or cannot be met at the appropriate level of performance by an equivalent device available on the market; (e) the health institution provides information upon request on the use of such devices to its competent authority, which shall include a justification of their manufacturing, modification and use; (f) the health institution draws up a declaration which it shall make publicly available, including: i) the name and address of the manufacturing health institution, ii) the details necessary to identify the devices, iii) a declaration that the devices meet the general safety and performance requirements set out in annex I to this regulation and, where applicable, information on which requirements are not fully met with a reasoned justification therefor; (g) as regards class D devices in accordance with the rules set out in annex VIII, the health institution draws up documentation that makes it possible to have an understanding of the manufacturing facility, the manufacturing process, the design and performance data of the devices, including the intended purpose, and that is sufficiently detailed to enable the competent authority to ascertain that the general safety and performance requirements set out in annex I to this Regulation are met. member states may apply this provision also to class A, B or C devices in accordance with the rules set out in annex VIII; (h) the health institution takes all necessary measures to ensure that all devices are manufactured in accordance with the documentation referred to in point (g); and (i) the health institution reviews experience gained from clinical use of the devices and takes all necessary corrective actions. Member States may require that such health institutions submit to the competent authority any further relevant information about such devices which have been manufactured and used on their territory. Member States shall retain the right to restrict the manufacture and use of any specific type of such devices and shall be permitted access to inspect the activities of the health institutions. This paragraph shall not apply to devices that are manufactured on an industrial scale.


B| Transitional Provisions^21^^,^^22^
Purple: applicable since 26 May 2022 Green: applicable since 26 May 2024 Orange: shall apply from 31 December 2030

### Article 5(5) of EU-IVDR

Recital #29 of the EU-IVDR emphasizes the special importance of health institutions and the IVDs developed by them. Accordingly, the development, manufacture and use of in-house tests by health institutions should continue to be an option to meet the specific needs of patients. For this purpose, however, recital #28 states that the rules governing IVDs, manufactured and used within a single health institution only, should be clarified and strengthened to ensure the highest level of health protection.^3^ In line with this recital, the EU-IVDR specifies a uniform EU-wide approach in Article 5(5): IVDs that are manufactured and used solely within health institutions established in the Union, and that are not manufactured on an industrial scale, are exempt from most of the provisions of the EU-IVDR, provided that the health institution complies with Article 5(5).^3^ To ensure the highest level of health protection, Article 5(5) sets a number of conditions regarding the manufacture of such in-house IVDs (Table 3A).^23^

A key aspect is that IH-IVDs must meet the relevant general safety and performance requirements (GSPR) set out in Annex I of the EU-IVDR [Article 5(5), sentence 1]. This means that health institutions must ensure and demonstrate that (i) IH-IVDs are safe for patients and users, (ii) potential risks are known and controlled, (iii) performance is evaluated and (iv) that the IH-IVDs are fit for their predefined intended purpose.^24^ EU-IVDR stipulates that manufacture and use of IH-IVDs must occur under appropriate quality management systems and that the laboratory of the health institution is compliant with standard EN ISO 15189 or, where applicable, with national provisions [Article 5(5) (b) and (c)]. Further, EU-IVDR stipulates an extensive documentation regarding the manufacturing facility, the manufacturing process, the design and performance data of the devices, and the intended purpose. This is meant to enable national competent authorities to ascertain that the GSPR set out in Annex I are met [Article 5(5) (g)] by inspecting health institutions. Compliance with the GSPR must be publicly declared [Article 5(5) (f)]. (Table 3A and B).

To provide guidelines supporting the implementation of the EU-IVDR, the Medical Device Coordination Group (MDCG) was established in accordance with Articles 98 and 99 of the EU-IVDR. This group comprises representatives from all EU member states and is chaired by a European Commission representative. In 2023, this group published a general basic guidance document on the health institution exemption under Article 5(5) of the EU-IVDR.^4^ There are also more specific guidance documents and templates from various professional societies and disciplines.^24^, ^25^, ^26^, ^27^ These guidance documents are not legally binding and merely serve as a proposal for implementing the EU-IVDR requirements for health institutions.

### Outlook and perspective

Since the introduction of the EU-IVDR, there have been many critical discussions among various stakeholders including national and European professional associations.^28^, ^29^, ^30^, ^31^, ^32^ There have also been several amending regulations that have repeatedly postponed conditions and individual articles (e.g. due to the COVID-19 pandemic and due to a perforce gradual roll-out of the European database on medical devices, which shall provide an up-to-date overview of the lifecycle of medical devices that are made available in the EU).^21^^,^^22^^,^^33^^,^^34^

In accordance to Article 111, by 27 May 2027, the European Commission shall assess the application of the EU-IVDR and prepare an evaluation report on the progress made toward achieving the objectives including an assessment of the resources required to implement the Regulation. In 2024, the European Commission’s initiative to evaluate the EU-IVDR was brought forward although not all conditions are in force yet.^35^ In addition, the European Parliament submitted a joint motion for a resolution on the urgent need to revise the EU-IVDR.^36^ The purpose of the targeted evaluation is to help the Commission to assess whether the rules are effective, efficient and proportionate; whether they meet current and emerging needs; whether they are consistent with other actions; and whether they provide added value.^35^

## Similarities and differences in considerations regarding the regulation of ldts in the USA and IH-IVDS in the EU

As shown in Figure 1, there are key regulatory divergences of in-house diagnostics regulated in the USA and in the EU. In both jurisdictions, there are strong arguments for incorporating LDTs and IH-IVDs used in medical laboratories and health care institutions into regulatory frameworks. The fact that both jurisdictions have specific, albeit different, requirements for LDTs and IH-IVDs demonstrates the importance of these tests in diagnostics.^37^ Table 2 lists selected core regulatory parameters and provides a comparative overview of their required implementation.

The FDA argued that LDTs are becoming ‘increasingly complex’ and are produced in higher volumes for larger and more diverse populations, meaning the ‘associated risks are also growing in complexity and impact’.^14^ EU legislation emphasized that the rules governing IH-IVDs should be clarified, harmonized and strengthened to ensure the highest level of health protection (Recital #28 of EU-IVDR).

Although it is undisputed that patient and user protection are paramount when using self-developed tests, the type and manner of the regulatory framework, the definition of the scope of application, the exact need for regulation and the extent and effort involved are subject of dispute.

### Considerations and concerns regarding the regulation of LDTs in the USA

The final judgment of the recent lawsuit against the FDA’s Final Rule and the lack of an appeal brings clarity to the regulation situation in the USA. The FDA’s Final Rule exceeds its authority and is unlawful.^1^ The FDA does not have the authority to regulate LDTs as medical ‘devices’ until there is no explicit legislative guidance. Before this final judgment, there were several stakeholder consultations to address considerations and concerns regarding the regulation of LDTs in the USA. Following a stakeholder call by the FDA after publication of its proposed rule, around 6700 comments were submitted.^38^ Additionally, the Health Subcommittee of the House Energy & Commerce Committee of the United States House of Representatives held a hearing entitled ‘Evaluating Approaches to Diagnostic Test Regulation and the Impact of the FDA’s Proposed Rule’ in March 2024, which included testimony from key stakeholders.^39^, ^40^, ^41^, ^42^, ^43^ ACLA was concerned that the FDA’s rule will reduce patients’ access to laboratory testing services, as laboratories will have to remove services from their testing menus due to the high cost of FDA approval.^42^ They consider that the rule formally exceeds the FDA’s jurisdiction, because laboratory developed testing services are not devices and the FDA lacks authority to regulate them as such. CAP mentioned the importance of LDTs because they are typically developed for patients with rare diseases or to meet specific clinical needs. CAP believes that a legislative and regulatory framework for LDTs should include a role for the CLIA and FDA, according to a test’s risk level to a patient, but duplications in regulatory requirements must be avoided.^41^ Accordingly, CAP calls for a revision and modification of the FDA rule in the manner of the aforementioned VALID Act. Also, the Academic Coalition for Effective Laboratory Developed Tests and AMP stated that LDTs are not devices under the FDA’s definition, and rejects the FDA’s rule. However, they also called for modernizing CLIA and the need for regulatory advancements focused on enhanced proficiency testing in line with laboratory science.^39^ In contrast, AdvaMedDx, a division of the Advanced Medical Technology Association, supported the FDA’s proposed oversight on LDTs.^43^ They emphasized the current fragmented regulatory landscape for diagnostic testing and supported the FDA ruling to create clarity and uniformity within a risk-based and flexible framework. They argued that CLIA oversight focuses on laboratories, paying attention to their overall laboratory operations and performance, but not on LDTs, which are ‘devices’ in AdvaMedDx’s point of view.^43^

### Considerations and concerns regarding the regulation of IH-IVDs in the EU

As part of the ongoing evaluation of EU-IVDR, there is a call for evidence gathered through stakeholder consultation, literature review, annual reports of national competent authorities and relevant studies. A public consultation was open from December 2024 to March 2025 and is publicly available.^44^ A factual summary report published in June 2025 provides an overview of the number of responses received and the characteristics of the respondents. It also summarizes some of their views and concerns.^45^ A summary report of the stakeholder’s responses shall be published, and a synopsis report shall accompany the final evaluation report. Adoption by the Commission is planned for the fourth quarter of 2025. Very recently, the European Commission published an additional initiative for targeted revision that aims to ‘streamline and future-proof the regulatory framework by reducing the administrative burden and enhancing predictability and cost-efficiency, while preserving a high level of public health and patient safety, and thus contribute to the initial objectives of the Regulations’.^46^ A call for evidence was issued during the feedback period from 8 September to 6 October 2025. Although the previous targeted evaluation was focused on identifying problems in the legal framework, this call for evidence aims to gather input to address the identified problems in a targeted way.^46^ However, even before these measures were taken, various stakeholders, including professional societies, health care institutions and industry representatives, have published different opinion papers on the regulation of IH-IVDs. Worth mentioning, for example, are the statements and findings of the university hospitals of Leuven (Belgium) and Oslo (Norway), as well as the joint declaration by German professional associations of diagnostic disciplines, which refer directly to Article 5(5) and call for a reduction in time-consuming administrative burdens.^47^, ^48^, ^49^ Many national and Europe-wide medical professional societies fully support the EU-IVDR’s intent to raise quality and transparency in the *in vitro* diagnostics field, but they refer to the already existing robust quality assurance systems. However, it is also clarified that the manufacture of IH-IVDs did not fall within the scope of the previous regulation and that this gap can therefore be closed by the EU-IVDR, although changes and a reduction in bureaucracy are necessary.^28^^,^^31^^,^^49^, ^50^, ^51^, ^52^, ^53^ The Biomedical Alliance in Europe (BioMed Alliance), an initiative of leading European medical societies, argues that EU-IVDR’s restrictions on in-house tests are too stringent and risk stifling innovation in oncology. Although hospital laboratories’ assays can fill critical gaps, especially for rare biomarkers or urgent research needs, these stakeholders consider that the EU-IVDR discourages their development. The continued use of well-validated in-house tests should be granted, even if a similar commercial test exists, with appropriate quality standards. They encourage regulators to trust accredited laboratories and enable flexibility (e.g. by permitting inter-laboratory sharing of tests and clear guidelines rather than outright prohibitions).^31^^,^^54^ Among other professional societies, the European Society for Medical Oncology (ESMO), European Cancer Organisation, the European Alliance for Personalized Medicine, European Federation of Clinical Chemistry and Laboratory Medicine, BioMed Alliance and industry have consistently pointed out that certain unintended consequences of the EU-IVDR – delays in clinical trials, potential loss of tests, and administrative hurdles for innovation – pose real risks to oncology care and research in Europe. Through detailed position papers and collective advocacy, these organizations have shone a light on these issues and offered practical solutions such as extended transition timelines, increased regulatory capacity, harmonized processes, protected laboratory-developed innovation and aligned diagnostic approvals with treatment advances.^31^^,^^54^, ^55^, ^56^, ^57^, ^58^, ^59^, ^60^

The guidance document MDCG-2022-10 focuses on the interface between Regulation (EU) 536/2014 on clinical trials for medicinal products for human use and EU-IVDR, including the use of IH-IVDs in clinical trials.^61^ Assays used in clinical trials may range from CE-IVDs to trial- or medicinal product-specific assays that can be IH-IVDs. All IVDs used in clinical trials must comply with EU-IVDR, meaning that IH-IVDs must meet the conditions set out in Article 5(5). Article 5(5) (d) stipulates that an available CE-certified companion diagnostic (CDx) must be preferred to an equivalent IH-IVD. This creates a preference for centralized testing via IVDs that weakens academically driven innovation and methodological freedom in molecular diagnostics and consequently slows down adaption to patient needs. As samples are pretested locally on site under quality assured conditions in accordance with Article 5(5), the current regulation leads to duplicate tests, longer analysis times (also due to shipment of material for central testing by a CDx, at least in phase III clinical trials), among others, all of which are at the expense of the patient.

The umbrella organization of the scientific medical societies in Germany [Association of the Scientific Medical Societies in Germany (AWMF)] further argues that IH-IVDs, because they are developed by a health care provider and are only used by this institution, are neither marketed nor made available on the EU market and should therefore not be in the scope of EU-IVDR.^28^ AWMF also stated that in accordance with Article 168 of the Treaty on the Functioning of the EU, responsibility for medical care lies with the EU member states. Consequently, the regulation of medical services should fall under national law and, as in Germany for example, also within the scope of self-administration of the health care system.

## Discussion

As LDTs and IH-IVDs become increasingly complex, it is understandable and expected that the regulatory frameworks governing in-house manufacture and clinical use evolve accordingly. This evolution reflects not only the scientific advances but also the heightened need for transparency, patient safety and reproducibility in clinical diagnostics.

In the EU, the EU-IVDR has replaced the longstanding Directive 98/79/EC on *in vitro* diagnostic medical devices. For the first time, although primarily aiming at regulating IVDs, it extends regulatory oversight to IVDs that are manufactured and used exclusively within health institutions. Although this shift to a single legal framework governing both IVDs and IH-IVDs aims to harmonize and raise quality standards across the EU, it also introduces a level of complexity that poses a challenge for many laboratories.

In contrast, there are two separate regulatory frameworks in the USA, one for LDTs and one for IVDs. LDTs are subject to supervision by the CMS via CLIA, for which there is no equivalent at the supranational EU level. The ‘competent authorities’ designated under the EU-IVDR come closest to a CLIA-like scenario, but their purpose and legal framework obviously differ significantly from those of CLIA. In our view, the dual approach pursued by the USA allows for better consideration of the specific circumstances and nature of LDTs and their use in multi-step laboratory processes. Whether a CLIA equivalent at the EU level is a meaningful option, or whether a separate, possibly delegated, regulation of IH-IVD at national level is preferable, should be discussed among relevant stakeholders. In any case, the current design of the EU-IVDR, which regulates IVD and IH-IVD in a single law, does not allow for adequate regulation of IH-IVD. Rather, the amalgamation of both regulatory areas creates an industry privilege [Article 5, 5 (d)] that leaves the issue of patient safety primarily to the market rather than to the treating physician, who bears full medical and legal responsibility for the diagnosis. Whether this is in line with the spirit of the law and the intention of the legislation should be clarified.

Notably, across both regions, the regulation of IH-IVDs and LDTs has become a focal point of policy and professional concern. Driven by the escalating complexity and clinical relevance of these tests, the regulatory expectations and associated conformity assessments are becoming increasingly intricate—and for many health care professionals and laboratory leaders—difficult to interpret or apply in practice. These developments raise important questions about proportionality, sustainability and the future of diagnostic innovation within health systems.

### Definition ambiguity and its consequences

**Figure 2:**
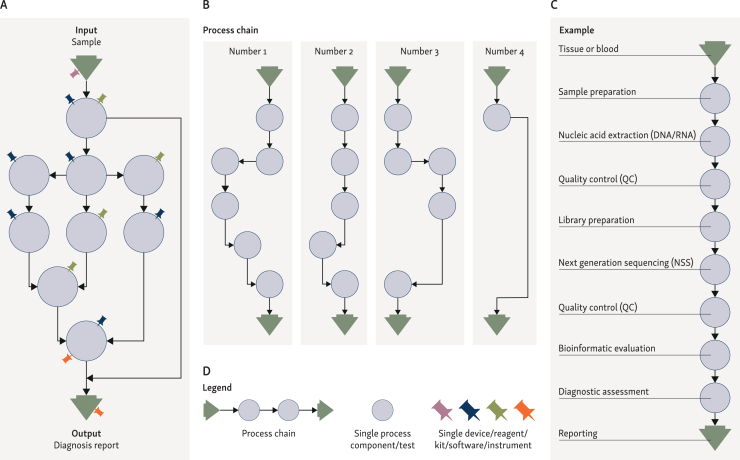
**What is an LDT/IH-IVD?** (A) Exemplary but typical network of process chains in diagnostic laboratories with branches and interconnections. Diverse IVDs (symbolized as a pin) can be involved in each step, either separately or in combination. (B) Splitting up the individual process chains is a common tool to validate diagnostic processes. It is usually not expedient to validate single devices/IVDs individually, since the laboratories must have the processes components or the whole processes-chain under control. (C) Exemplary process chain. (D) Legend. IH-IVD, in-house *in vitro* diagnostic medical device; LDT, laboratory-developed test.

It is difficult for both regulators and laboratories to clearly distinguish between an LDT or IH-IVD as (i) an entire process chain, (ii) a process component, (iii) a single device or as (iv) a medical service due to the interconnected and interdependent nature of meshed analytical steps of a diagnostic process (Figure 2).

Article 2 of the EU-IVDR defines ‘*in vitro* diagnostic medical devices’ (Table 1) the subject matter of the IVDR. This definition does not include complex analytical workflows in the medical laboratory, in line with the United States’ case law on the term ‘device’. However, the document MDCG-2023-01 Guidance Document on the health institution exemption under Article 5(5) provides a definition for an ‘in-house device’ as ‘a device that is manufactured and used only within a health institution established in the Union and that meets all conditions set in Article 5(5) of the […] IVDR’ (Table 1). Any product or a combination of products that meets the definition of ‘device’ must comply with the EU-IVDR [i.e. either be CE marked or be manufactured in-house by a health institution and thus comply with Article 5(5)]. The MDCG-2023-01 document also explicitly states that a laboratory protocol or laboratory results are not considered devices. This clearly indicates that the entire and complex diagnostic process chain required to make a definitive diagnosis is not considered to be an IH-IVD.

A further MDCG document is under development that provides guidance on ‘research use only’ devices used in diagnostic process chains under the EU-IVDR.^62^ The term ‘research use only’, which is claimed on these devices, has not yet been legally defined in the EU. It will therefore be interesting to see how this upcoming guidance deals with the use of these devices in diagnostic processes. Of note, these guidance documents are not legally binding; only the Court of Justice of the EU can give binding interpretations of Union law.

In the USA, the recent final judgment in favor of the plaintiffs to vacate and set aside the FDA’s Final Rule in its entirety confirms the notion that an LDT as a diagnostic process or as a medical service cannot be regulated by the FDA’s Final Rule.^1^^,^^2^ As a consequence, the FDA has jurisdiction over medical devices, but not over medical services in the form of LDTs or LDPs. According to AMP, one of the plaintiffs, this outcome highlights the important part played by clinical laboratories in developing and delivering innovative, high-quality diagnostics and recognizes the current regulatory framework within which these tests have been operated.^63^

Analogously, in Europe, it could be assumed that EU-IVDR regulates only tangible ‘devices’ and not laboratory processes as a whole, which are the responsibility of physicians. However, the Union legislator implicitly classifies diagnostic procedures that are manufactured and used exclusively within health institutions as IH-IVDs.^64^ In the absence of explicit clarification or legal judgment, diagnostic laboratories are currently left to operate under a broad and potentially inconsistent interpretation of this classification. This ambiguity underscores the need for clearer and specific regulatory guidance to avoid misapplication or unintended quality constraints on laboratory-developed procedures.

### Implications for laboratory practice and accreditation

Since it is more expedient for laboratories to validate processes than individual IVDs, they tend to define processes as IH-IVDs and combine several components of a process chain (Figure 2B). This comprehensive approach is essential for quality assurance of the service and its processes. This applies to (i) pre-analysis; (ii) the entire wet-laboratory workflow within the laboratory; (iii) subsequent post-analysis, bioinformatics pipelines; and (iv) the evaluation and reporting of findings. This strategy also considers critical interfaces between the individual process components and is part of an already existing robust quality assurance system that has been in place long before the EU-IVDR enforcement in Europe and is part of the CLIA oversight in the USA.

**Figure 3:**
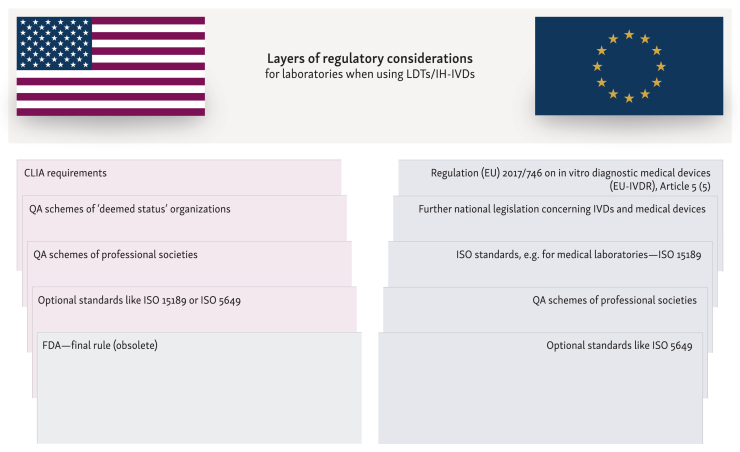
**Layers of regulatory considerations for laboratories when using LDTs/IH-IVDs.** CLIA, Clinical Laboratory Improvement Amendments; FDA, Food and Drug Administration; IH-IVD, in-house *in vitro* diagnostic medical devices; ISO 15189, ‘Medical laboratories - requirements for quality and competence’; ISO 5649, ‘Medical laboratories - concepts and specifications for the design, development, implementation, and use of laboratory-developed tests’; ISO, International Organization for Standardization; LDT, laboratory developed test; QA, quality assurance.

Compliance with accreditation standards, such as EN ISO 15189, which is also a condition of Article 5(5) of the EU-IVDR, means that all processes leading to diagnosis within laboratories must be validated, including the use of IH-IVDs within the validated process chains. EN ISO 15189 requirements for verification and validation of diagnostic tests are similar to those of the CLIA. Additional EN ISO 15189 certification is optional for United States laboratories. With compliance to EN ISO 15189 and with further national legal regulations, guidelines and quality assurance initiatives based on professional and medical associations, a very high level of patient safety is already in place (Figure 3).^65^

Like CLIA requirements, EN ISO 15189 does not regulate manufacture and lifecycle of single IH-IVDs as tangible medical devices. Legislators in the EU and USA have recognized the need for regulation in this area. Another new published international standard for medical laboratories is ISO 5649 ‘Medical laboratories - Concepts and specifications for the design, development, implementation and use of laboratory-developed tests’, which was introduced in 2024.^66^ This standard aims to globally standardize essential specifications for LDTs and IH-IVDs and sets out requirements for quality assurance, safety, performance and documentation of these tests. For this, it provides general and state-of-the-art practical support. However, there have been no documented experiences of its use so far. Use of this standard is voluntary and is not part of the current regulatory framework in either the EU or the USA.

### Jurisdictional tensions and the practice of medicine

Above all national laws, mandatory and optional standards as well as guidelines, the EU-IVDR, which intends to regulate both IVDs and IH-IVDs, represents a further layer of requirement that must be implemented on top of the existing and established internal laboratory quality assurance measures and in the quality management system (Figure 3). In the USA, CLIA certification provides a robust quality assurance system specifically designed for laboratory processes, even when LDTs are used within validated and established processes.

The recently published United States District Court decision highlights the difficulty in defining LDTs as tangible devices and the recent FDA initiative has failed for the time being due to a ruling in favor of the plaintiffs. In Europe, the targeted evaluation of the EU-IVDR is underway, and there are many statements and position papers from stakeholders to consider, including arguments similar to those made in the Memorandum Opinion and Order of the United States District Court.^1^ The recent position paper of the AWMF states that the performance of diagnostic procedures and laboratory medical examinations by physicians and under their responsibility must be regulated at EU member state level in accordance with Article 168 of the Treaty on the Functioning of the EU and that Article 5(5) of the EU-IVDR is therefore contrary to the Treaty. It must be the physician’s responsibility to choose the most ethically and medically appropriate method to meet the patient’s need.^28^ This view is similar to the FD&C Act statement that ‘practice of medicine’ is outside of the FDA’s jurisdiction, which the United States District Court relied on as part of its ruling (Sec. 396 - Practice of medicine).

### Prospects for harmonization

A fundamental challenge in regulating laboratory diagnostics lies in the conceptual misalignment between regulatory frameworks and real-world laboratory practice. Regulatory systems—both in the USA and the EU—are primarily device-focused, built to assess and control tangible IVDs. In contrast, diagnostic laboratories operate with process-oriented logic, relying on interconnected procedures and interpretative workflows that extend beyond the scope of individual devices (Figure 2). It is therefore important to distinguish between a ‘test’ (a discrete analytical component), a ‘process’ (the structured workflow involving multiple analytical steps), and a ‘service’ (the complete diagnostic output including clinical interpretation and consultation). This distinction has been formalized in a diagnostic quality system,^67^ which emphasizes the need to evaluate laboratory performance across these interconnected layers rather than at the level of isolated devices. The lack of alignment between these operational realities and existing regulatory constructs creates regulatory friction, particularly in the validation and oversight of laboratory-developed and in-house diagnostics.

As such, it may be that a practical, scientific discussion by those routinely involved in the delivery or use of these tests (e.g. the AMP, American Society of Clinical Oncology, European Society of Pathology, ESMO), governed by two driving principles (the need of the highest test quality within the most practical delivery framework), may help regulatory agencies across the Atlantic to (i) unify concepts and (ii) harmonize criteria.

### Conclusion

As regulatory frameworks for LDTs and IH-IVDs evolve, clarity, proportionality, and international coherence must guide future policy. Stakeholders across jurisdictions should prioritize alignment between regulatory expectations and laboratory realities to safeguard innovation and patient safety. We hope that this overview of the complexities between EU and USA regulation around diagnostic tests can help policymakers, laboratories, and stakeholders better navigate current regulatory frameworks, anticipate future changes, and contribute to more coherent and patient-centered diagnostic oversight. A specific effort to unify concepts and harmonize criteria, balancing innovation and safety, may go a long way to provide a better care to our European and American patients.
